# EphB4 and ephrin-B1 expression in the intra-testicular-resident macrophages in mice

**DOI:** 10.5455/javar.2024.k826

**Published:** 2024-09-29

**Authors:** Md. Royhan Gofur, Kazushige Ogawa

**Affiliations:** 1 Department of Veterinary and Animal Sciences, University of Rajshahi, Rajshahi, Bangladesh; 2 Laboratory of Veterinary Anatomy, Graduate School of Veterinary Science, Osaka Metropolitan University, Izumisano, Osaka, Japan

**Keywords:** EphB4, ephrin-B1, F4/80, intratesticular-resident macrophages, tyrosine-phosphorylation

## Abstract

**Objective:**

The objective was to find out the expression of EphB4 receptor and ephrin-B1 ligand by the macrophages that live inside the mouse testicles.

**Materials and Methods:**

Messenger ribonucleic acid (mRNA) expression of EphB4 and ephrin-B1 was identified via RT-PCR amplification, and protein expression was examined by immunostaining.

**Results:**

Analysis using RT-PCR revealed that mRNA of EphB4 and ephrin-B1 were noticed in the examined testis of all postnatal ages. Furthermore, immunostaining revealed that F4/80-positive intra-testicular-resident macrophages were located in the intertubular spaces within the testis and more densely around the intra-testicular excurrent duct system, and increased in number gradually during the postnatal period of development until 5 weeks of age, when the mice attain their maturity (puberty), and maintained thereafter. Both EphB4 and ephrin-B1 immunoreactivity were noticed in F4/80-positive intra-testicular-resident macrophages within the testis of all studied postnatal ages. Ephrin-B1 and EphB4 immunoreactivity were weak during early postnatal development until the age of 2 weeks, and then ephrin-B1 immunoreactivity became very strong and EphB4 immunoreactivity became strong at the age of 3 weeks, and they continued to do so until the age of 8 weeks. Furthermore, EphB4 receptor was tyrosine-phosphorylated in testis.

**Conclusion:**

The expression of EphB4 and ephrin-B1 in mice intra-testicular-resident macrophages is being examined for the first time in this work. The localization of EphB4 and ephrin-B1, and EphB4 tyrosine-phosphorylation suggest that EphB4/ephrin-B1 signaling might occur in the intra-testicular-resident macrophages, and may participate in maintaining male fertility.

## Introduction

The testis is a special immunological milieu where inflammatory reactions are often inhibited that shields gametes from immune system assault and maintains fertility. A key element of this specific immunological milieu is that the testicular macrophages (Mø) have a major role in immunological function in the testis [1,2] and encompass a substantial fraction of the testicular interstitial tissue [3]. The presence of Mø in the testis is necessary for normal spermatogenesis [4]. It is reported that Mø supports testicular functioning [5]. Testicular Mø are abundant near spermatogonial progenitors (undifferentiated spermatogonia) and express factors essential for spermatogonial differentiation [6], and also promote Leydig cell steroidogenesis [5].

Macrophages can be broadly categorized into two types: tissue-resident and recruited Mø. Tissue-resident macrophages exhibit remarkable heterogeneity and phenotypic uniqueness [7]. These macrophages settle in organ- and tissue-specific microenvironments (the Mø niche) that support steady-state long-term survival. They then maintain tissue/organ homeostasis by acting in a way that is distinct to each tissue and organ [4,8]. The niche gives Mø nutrition, cytokines, and signaling molecules, allowing them to self-renew and take on characteristics unique to particular tissues or organs [8]. Recruited Mø, on the other hand, comes from BMMs that enter lesions as a reaction to tissue/organ damage and inflammation to resolve them.

The membrane proteins Eph and ephrin function as likely a system of connection between cells. Considering the homology of their extracellular domain amino acid sequences, Eph receptors in mammals are classified into two classes: EphA (A1–A8 and A10) and EphB (B1–B4 and B6). EphA and EphB receptors randomly bind to the ephrin-A (A1–A5) and ephrin-B (B1–B3) ligands, respectively [9]. It has been thoroughly studied how Eph and ephrins function in the growing tissues, particularly in the vascular and central neurological systems, where they control blood vessel maturation, tissue-border development, axon guidance, and cell migration [9,10]. In recent times, it has been observed that EphB and ephrin-B play critical roles in normal physiology and balance of tissues and organs, including maintaining homeostasis and epithelial boundary formation in several epithelia likely in the gut, stomach, mammary glands, skin, and male genital excurrent duct system [11–13]. Very recently, the expression of EphB4 and ephrin-B1 was found in spermatogonia, steroidogenic cells in the testis (Leydig cells) [12,13], and the ovary (theca cells, granulosa cells, and luteal cells) [14]. As tissue-resident Mø colonizes around steroidogenic cells in gonads and uterus [6,15], we hypothesized that EphB4 and ephrin-B1 may express in testicular macrophages. So, we looked at the EphB4 receptor and ephrin-B1 ligand expression in mouse intra-testicular resident macrophages in this work.

## Materials and Methods

### Animals and ethical approval

One day, 1, 2, 3, 4, 5, 6, and 8-week ICR mice, housed in conventional housing and food settings, were employed for RT-PCR, immunoblotting, and immunohistochemical studies on their testes. Testis samples for RT-PCR were obtained from mice that were 1 day and 1 week of age (*n =* 6 per group; two mice’s tissues were combined to create a single sample), as well as from mice that were 2, 3, 4, 5, 6, and 8 weeks of age (*n =* 3 per group; one mouse is for each sample). We examined the samples (testis) from three mice of each phase of development for immunohistochemistry. Mice classified as neonatal were those that were 1 day to 1 week old, while prepubertal, pubertal, and adult mice were characterized as those that were 3–4 weeks, 5–6 weeks, and 8 weeks old, respectively. The guidelines for using animals in experiments were permitted by the Osaka Metropolitan University Animal Research Committee (approval number: 29-12).

### Antibodies

The antibodies utilized in this investigation are enumerated in Table 1.

### RT-PCR analysis

Utilizing TRIzol reagent (Invitrogen, USA), total mRNA was extracted from mice testis that were one day, 1, 2, 3, 4, 5, 6, and 8 weeks old.

**Table 1.** An inventory of used primary and secondary antibodies with their supplier company.

Name of antibody	Name of supplier company
Goat anti-mouse EphB4 and ephrin-B1 polyclonal antibody	R&D Systems, Inc. (Minneapolis, MN, USA)
Rabbit anti-mouse ephrin- B1/2/3 polyclonal antibody	Santa Cruz Biotechnology (Dallas, TX, USA)
Rat anti-mouse F4/80 monoclonal antibody	BMA Biomedicals (Augst, Switzerland)
Alexa Fluor 488-conjugated donkey anti-goat IgG Alexa Fluor 488-conjugated donkey anti-rabbit IgG Alexa Fluor 594-conjugated donkey anti-rat IgG	Molecular Probes, Inc. (Eugene, OR, USA)
Horseradish peroxidase (HRP)-conjugated anti-phosphotyrosine antibody (PY20)	BD Transduction Laboratories (San Jose, CA, USA)
HRP-conjugated mouse anti-goat IgG	Jackson ImmunoResearch Laboratories, Inc. (West Grove, PA, USA)

An analysis using RT-PCR was then employed following the technique described earlier [12]. Briefly, 1 μg of total RNA was then converted into first-strand complementary deoxyribonucleic acid (cDNA) by employing oligo (dT)_18 _primer, RNase H− (Promega, USA), and M-MLV reverse transcriptase. As a template, we used reverse-transcribed cDNA, and a 0.5 μl reaction mixture was amplified with Taq DNA polymerase (TaKaRa Ex Taq HS; Takara Bio Inc., Japan) to determine endogenous EphB4, ephrin-B1, and β-actin. The primer pairs of the corresponding proteins and their cycle numbers that were utilized in PCR amplification for the current investigation are listed in Table 2. Following separation on 1.5% agarose gels, the PCR findings were made visible using ethidium bromide staining.

### Immunoprecipitation and immunoblotting

A modified radioimmunoprecipitation assay buffer was used to homogenize the testis devoid of tunica albuginea. Supernatants were gathered after 10 min of high-speed centrifugation, and a Protein Assay kit from Bio-Rad Laboratories in Hercules, California, USA, was used to quantify the protein concentrations.

Immunoblotting and immunoprecipitation procedures were followed as earlier mentioned [16]. In brief, for immunoprecipitation, after incubating 1,000 μg of tissue extracts with 1.5 μg anti-EphB4 or 0.5 μl normal goat serum (as a control; Vector) for a whole night at 4°C, 15 μl protein G magnetic beads (Thermo Scientific, Waltham, MA, USA) were applied for 1 h at 4°C. Separating the immunoprecipitate on 10% polyacrylamide gels, the membrane was then coated with polyvinylidene fluoride and incubated at 4°C for a whole night in Tris-buffered saline containing 0.1% Triton X-100 (TBS-T), 3% bovine serum albumin (BSA), and 1:5,000 HRP-conjugated PY20. A chemiluminescence reagent called ECL Prime (Amersham Biosciences, Uppsala, Sweden) was used to generate the immunoblot. Using 0.15 μg/ml anti-EphB4 antibody in TBS-T with 3% BSA and 0.2% non-fat dry milk, the membrane was reprobed. The immunoblot was produced once more following incubation with 1:20,000 HRP-conjugated mouse anti-goat IgG.

**Table 2.** The cycle numbers and primer pairs used in PCR amplification.

Primer			Product size (bp)	Annealing temp. (°C)	Cycle number
EphB4	Forward	5´-AGCCCCAAATAGGAGACGAG-3´	540	57.9	29
	Reverse	5´-GGATAGCCCATGACAGGATC-3´			
ephrin-B1	Forward	5´-TGCTTGATCCCAATGTACTG-3´	520	55.0	29
	Reverse	5´-CGGAGCTTGAGTAGTAGGAC-3´			
β-actin	Forward	5´-TCATGAAGATCCTGACCGAG-3´	312	47.0	21
	Reverse	5´-GGTCTTTACGGATGTCAACG-3´			

**Table 3.** Tissue sample fixation protocols for immunohistochemistry.

Mice age	Fixation time	Washing time	Soaking time in 30% sucrose solution	Amount of fixative/tissue
1d	2 h	1 h	3.5 h	1 ml
1w	2 h	1 h	4.5 h	1 ml
2w	3.5 h	1 h	7 h	10 ml
3w–8w	4 h	1 h	Overnight	10 ml

### Immunofluorescence staining

The mouse testis samples were preserved in 10% formalin in phosphate buffered saline (PBS) at 4°C for various times at the various ages displayed in Table 3. Following PBS washing, submerged for 3.5 h to overnight in 30% sucrose solution in PBS (Table 3), after which embedded in the optimal cutting temperature compound. Next, fluorescent staining was applied to cryostat sections that were 5 μm thick.

Immunofluorescence staining was executed as mentioned earlier [12]. In summary, in a humid chamber, the 5 μm thickened cryostat sections were treated with 1% BSA in PBS and then incubated with primary antibodies at a concentration of 0.5 μg/ml anti-F4/80, 1 μg/ml anti-ephrin-B1, 1 μg/ml anti-ephrin-B1/2/3, and 4 μg/ml anti-EphB4 at 32°C for 1.5 h. Following a PBS wash, incubated the selected sections with secondary antibodies at a concentration of 5 μg/ml Alexa Fluor 488-conjugated donkey anti-goat Immunoglobulin G (IgG), 5 μg/ml Alexa Fluor 488-conjugated donkey anti-rabbit IgG, and 5 μg/ml Alexa Fluor 594-conjugated donkey anti-rat IgG in BSA-PBS for 0.5 h at 32°C. 4’, 6-diamidino-2-phenylindole dihydrochloride (1:500) was involved in the secondary antibody mixture to stain some sections to identify the nucleus. The PBS-washed sections were attached with PermaFluor and studied using a fluorescent microscope (IX71; Olympus, Japan).

## Results

### Expression of EphB4 and ephrin-B1 in postnatal developing and adult mice testis

**Figure 1.**
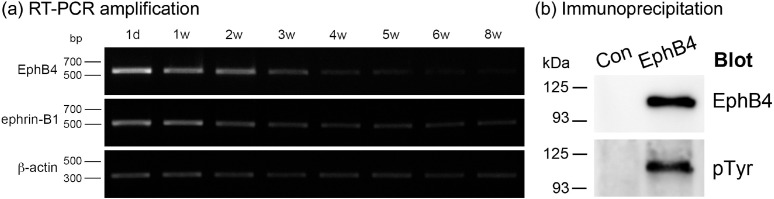
(a) RT-PCR amplification of EphB4 and ephrin-B1 mRNA from mouse testis of different postnatal ages. Transcripts of both proteins were noticed in the testis at all examined postnatal ages. (b) EphB4 in adult mouse testis was tyrosine phosphorylated. d, day; w, week; Con, Control; pTyr, tyrosine phosphorylation.

To determine whether the EphB4 and ephrin-B1 were present in the testis or not, mice testis of various postnatal ages were screened using RT-PCR. At every postnatal age studied, transcripts for both proteins (Fig. 1a) were found in the testis. Immunoprecipitation was employed for the recognition of EphB4 protein in adult testis. EphB4 protein was noticed by immunoprecipitation in adult testis. Moreover, EphB4 was tyrosine-phosphorylated (Fig. 1b), indicating that EphB4-expressing cells interact with ephrin-B1-expressing cells, and the receptor is triggered for forward signaling *in vivo* in the testis.

### Ephrin-B1 and EphB4 immunoreactivity in intra-testicular-resident macrophages in the postnatal developing and adult mice

It was previously discovered that in both postnatal developing and adult mice, spermatogonia and a subset of stromal cells of the testis, such as myoid flattened cells and Leydig cells, co-express EphB4 and ephrin-B1. Hence, in this instance, we aimed to investigate their expression in the macrophages that dwell inside the testicles of postnatally growing and adult mice. To do this, we used F4/80 immunostaining to identify macrophages inside the testis, as F4/80 is a major macrophage marker [17].

**Figure 2.**
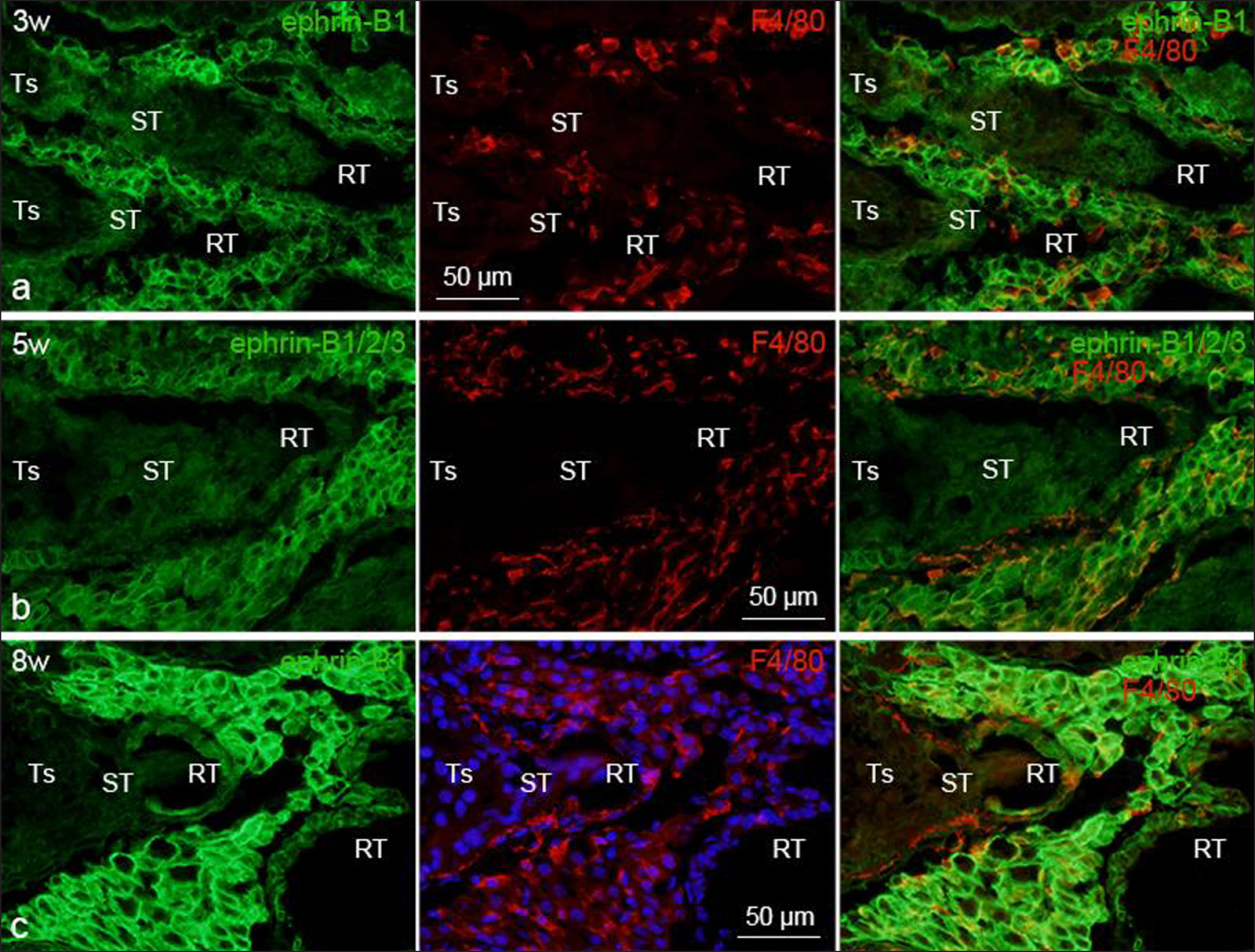
Immunofluorescence photomicrographs showing ephrin-B1 and/or ephrin-B1/2/3 expression in the intra-testicular-resident macrophages at different postnatal ages. The designated antibodies and/or DAPI were used to stain the sections. Double immunofluorescence staining of ephrin-B1/ephrin-B1/2/3 and F4/80 in the mouse testis of (a) 3 weeks (3w), (b) 5 weeks (5w), and (c) 8 weeks (8w) of age showing ephrin-B1 and/or ephrin-B1/2/3 stained the F4/80-positive intra-testicular-resident macrophages at all postnatal ages examined. RT, rete testis; Ts, seminiferous tubule; ST, straight tubule.

**Figure 3.**
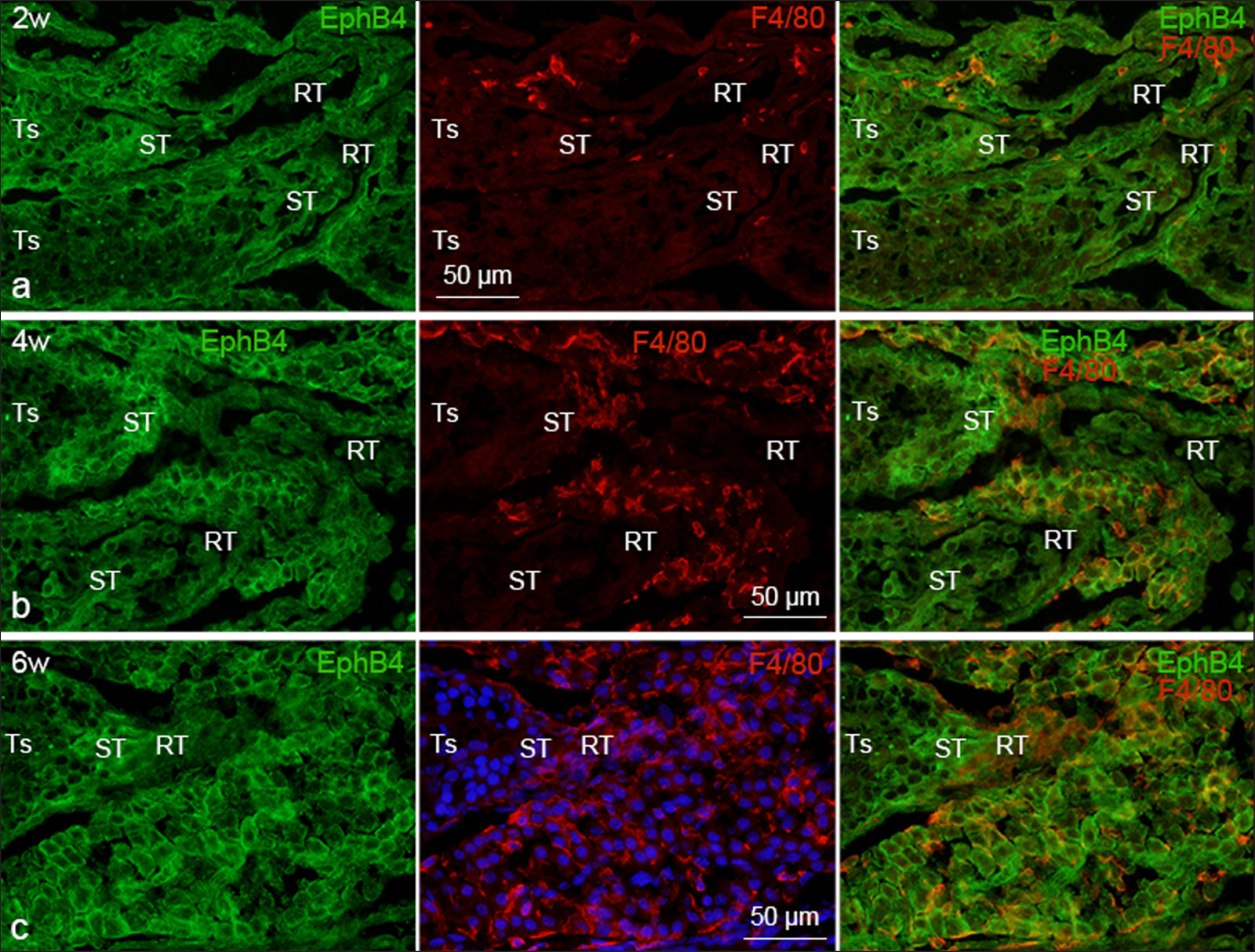
Immunofluorescence photomicrographs showing EphB4 expression in the intra-testicular-resident macrophages at different postnatal ages. The designated antibodies and/or DAPI were used to stain the sections. Double immunofluorescence staining of EphB4 and F4/80 in mouse testis of 2, 4 and 6 weeks of age showing EphB4 is expressed in F4/80-positive intra-testicular-resident macrophages at all postnatal ages examined. w, weeks; RT, rete testis; Ts, seminiferous tubule; ST, straight tubule.

F4/80-positive macrophages were located in the interstitial (intertubular) spaces within the testis, and more densely around the intra-testicular excurrent duct system (straight tubule and rete testis) (Figs. 2 and 3). It was observed that F4/80-positive intra-testicular-resident macrophages were increased in number gradually during the postnatal period of development until 5 weeks of age, when the mice attained their maturity (puberty), and maintained afterward (Figs. 2 and 3).

**Figure 4.**
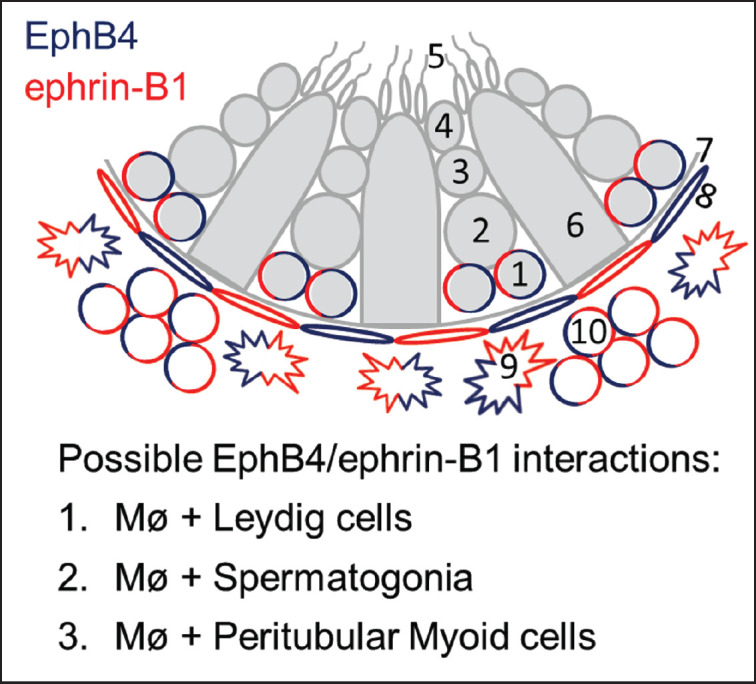
EphB4 and ephrin-B1 expression in intra-testicular-resident macrophages and their possible EphB4/ephrin-B1 interactions. 1, spermatogonia; 2, primary spermatocytes; 3, secondary spermatocyte; 4, spermatid; 5, spermatozoa; 6, Sertoli cell; 7, basement membrane; 8, peritubular myoid cell; 9, intra-testicular-resident macrophage (Mø); 10, Leydig cell.

Ephrin-B1 immunoreactivity was identified in F4/80-positive macrophages within the testis of all studied postnatal ages. Ephrin-B1 immunoreactivity in intra-testicular-resident macrophages was low throughout the first 2 weeks of postnatal development; however, it was highly expressed at 3 weeks and continued to be so until 8 weeks of age (Fig. 2). The F4/80-positive macrophages showed a comparable staining pattern when stained with ephrin-B1/2/3 antibody (Fig. 2b). Comparable to ephrin-B1, EphB4 immunoreactivity in intra-testicular-resident macrophages was also detected in F4/80-positive intra-testicular-resident macrophages in both postnatal developing and adult mice, and during the first 2 weeks of postnatal development, the expression intensity was weak. It then became strong at 3 weeks and remained that way until 8 weeks of age (Fig. 3). Moreover, comparing between EphB4 and ephrin-B1, the intensity of ephrin-B1 expression in intra-testicular-resident macrophages was stronger than that of EphB4 at all examined postnatal ages. The expression outline of EphB4 and ephrin-B1 in intra-testicular-resident macrophages and the possible EphB4/ephrin-B1 interactions are demonstrated in Figure 4.

## Discussion

Eph and ephrin function as a communication pathway between the cells and are responsible for facilitating repulsion or adhesion between adjacent cells, which in turn helps to establish tissue organization and development [9]. Eph/ephrin interaction leads to the propagation of bidirectional signaling. Phosphorylation by other tyrosine kinases, autophosphorylation, and the interaction of receptors with different effector proteins are the key drivers of forward signaling by Eph receptors; in contrast, the Src kinase family proteins perform a major role in the reverse signaling of ephrins [9,18,19]. On the other hand, the mammalian testicular interstitium, the area of testis outside the seminiferous tubules, is made up of various cells, including Leydig cells that secrete testosterone that facilitates the normal spermatogenesis [20], myoid flattened cells, which surround seminiferous tubules, interconnect Sertoli cells directly, and afford structural support [6], and immune cells, primarily intra-testicular-resident macrophages under normal circumstances, which are involved in spermatogonial differentiation [6]. The transcriptome of spermatogonial stem cells (SSCs) is enriched in genes related to immune cell differentiation and chemotaxis [21], indicating that communication between immune cells (like macrophages) and SSCs may be crucial for spermatogenesis [6]. DeFalco et al. [6] demonstrated that temporary macrophage depletion in testis does not upset SSC maintenance, but it does lead to a reduction in spermatogonia, possibly through an impact on spermatogonial differentiation. Moreover, testicular-resident macrophages are closely accompanying Leydig cells and support steroidogenesis by secreting 25-hydroxycholesterols and several cytokines [22,23]. It is reported that the depletion of testicular macrophages decreases testosterone secretion by Leydig cells [5,6,24]*. *Though intra-testicular-resident macrophages are involved in Leydig cell steroidogenesis and spermatogonial differentiation, the mechanism that mediates this interaction is not fully understood.

The analysis of RT-PCR expression of Eph4 and ephrin-B1 revealed that testis of all postnatal ages examined express these molecules. Moreover, our immunohistochemical analysis demonstrated that F4/80-positive intra-testicular macrophages in both postnatal developing and adult mice express both EphB4 and ephrin-B1. The present study is the first for EphB4 and ephrin-B1 expression studies in intra-testicular-resident macrophages, and our findings indicate that EphB/ephrin-B interaction might occur when testicular macrophages are in contact with cells expressing EphB and/or ephrin-B within the testis due to the promiscuous binding of EphB to ephrin-B within the same class [18]. Our Western blotting results, which showed that EphB4 was tyrosine-phosphorylated in the testis, partially corroborate this hint and imply that EphB4-expressing cells interacted with ephrin-B1-expressing cells within the testis.

Interstitial testicular-resident macrophages and Leydig cells have a close physical relationship, which may suggest a potential functional relationship between these cells. Under normal physiological circumstances, macrophages perform a vital role in Leydig cell development and functions [24]. Ultrastructural investigations demonstrated that intra-testicular resident macrophages form intercytoplasmic digitations with Leydig cells that physically connect the two cells, which are necessary for Leydig cell steroidogenesis [3,5,22]. Leydig cells, the major component of the testicular interstitium, express both EphB4 and ephrin-B1 [12,13]. Intra-testicular-resident macrophages co-localize among Leydig cells within testicular stroma [6,24,25] and also express EphB4 and ephrin-B1 detected in this study. This indicates that Leydig cells connect the intra-testicular-resident macrophages, and accordingly the EphB4/ephrin-B1 signaling arises from the interaction between them, and this speculation is supported by EphB4 tyrosine-phosphorylation in the testis. Moreover, intra-testicular-resident macrophages increased in number gradually during the postnatal period of development until 5 weeks of age, when the mice attained their maturity (puberty), indicating their connection in testosterone production. To ascertain the involvement of EphB4/ephrin-B1 interaction in testosterone synthesis, more research will be needed.

Spermatogonia (located close to the basal lamina), not the gonocytes and spermatocytes located far from the basal lamina of seminiferous tubules, express both EphB4 and ephrin-B1 [18,19]. Leydig cells, flattened myoid cells, and macrophages are all interstitial/stromal cells, and are regarded as cellular constituents of the SSC niche [3,26]. EphB/ephrin-B has been linked to bone marrow stem cell niche modulation [27]. EphB4 and ephrin-B2 are expressed by stromal cells and hematopoietic stem cells, respectively, and EphB4/ephrin-B2 signaling controls the expression of various cytokines to intercede hematopoiesis [28], and also the colonization and migration of the hematopoietic cells [29]. Therefore, it may be worthwhile to investigate whether EphB4/ephrin-B1 signaling, which is facilitated by interactions between spermatogonia and stromal cells, including intra-testicular-resident macrophages, is involved in SSC niche formation. A laminin-immunostaining investigation revealed that the basement membrane adjoining the seminiferous tubule is discontinuous in some areas [30]. In our previous study, it was observed that spermatogonia, not the gonocytes and spermatocytes, co-express EphB4 and ephrin-B1 [12,13]. We observed in the present study that intra-testicular-resident macrophages also co-express EphB4 and ephrin-B1, indicating spermatogonia likely contact the intra-testicular-resident macrophages, and therefore the EphB4/ephrin-B1 signaling rises from contacts between intra-testicular-resident macrophages and spermatogonia, and this speculation is supported by EphB4 tyrosine-phosphorylation in the testis. Moreover, a milieu that is favorable to spermatogonial differentiation into A1 spermatogonia may be formed by macrophages mediating the physical arrangement of peritubular flattened myoid cells in niche-like clusters [6]. However, to explain the role of the EphB4/ephrin-B1 interaction in SSC niche development, more research is required.

## Conclusion

This work provides the first examination of EphB4 and ephrin-B1 expression in intra-testicular resident macrophages in the normal testis of postnatal developing and adult mice. F4/80-positive macrophages were detected more densely around the intra-testicular excurrent duct system and increased in number gradually during the postnatal development until 5 weeks of age around puberty and maintained after that. We observed that both EphB4 and ephrin-B1 were expressed in intra-testicular resident macrophages and that the EphB4 receptor was tyrosine-phosphorylated and triggered for forward signaling in the testis. Immunoreactivity of both molecules was weak during early postnatal development until 2 weeks of age and then turned strong at the age of 3 weeks and persisted afterward until the age of 8 weeks. The results imply that the EphB4 receptor and the ephrin-B1 ligand are potential modulators of Leydig cell steridogenesis and spermatogonial differentiation, while further studies are needed to completely comprehend their roles.

## List of abbreviations

μm, micrometer; μg, microgram; ml, milliliter; °C, degree Celsius; h, hour; cDNA, complementary deoxyribonucleic acid; DAPI, 4’, 6-diamidino-2-phenylindole dihydrochloride; IgG, Immunoglobulin G; mRNA, messenger ribonucleic acid; PBS, phosphate buffered saline; OCT, optimal cutting temperature; bp, base pair; g, gravity; RIPA, radioimmunoprecipitation assay.
